# Endoscopic Resection of Right Petrous Meningioma Causing Trigeminal Neuralgia: “The Double Crush” Phenomenon

**DOI:** 10.1055/a-2599-4419

**Published:** 2025-05-21

**Authors:** Mazen Zaher, Pedro Aguilar-Salinas, Amna Hussein, Peter Nakaji

**Affiliations:** 1 Department of Neurosurgery, Banner University Medical Center, University of Arizona, Phoenix, Arizona, United States

**Keywords:** trigeminal neuralgia, cerebellopontine angle tumors, double crush phenomenon, microvascular decompression, endoscopic retrosigmoid craniotomy, neuropathic pain

## Abstract

Trigeminal neuralgia (TN) is typically caused by neurovascular compression (NVC) at the root entry zone, often involving the superior cerebellar artery. Occasionally, TN may be secondary to cerebellopontine angle (CPA) tumors, such as meningiomas, vestibular schwannomas, or epidermoid cysts. When both a tumor and a vascular loop contribute to nerve compression, the resulting, as we refer to the “double crush” phenomenon, complicates surgical management and necessitates a more comprehensive therapeutic strategy. Literature indicates that a simultaneous approach targeting both the tumor and the NVC is crucial to achieving optimal outcomes.

Microvascular decompression (MVD) alone may be insufficient for patients with tumor-associated TN, as the residual mass effect can persist. The literature suggests that combining MVD with tumor resection provides superior pain relief and reduces recurrence rates. An endoscopic retrosigmoid craniotomy offers enhanced visualization and maneuverability, allowing complete tumor resection and effective nerve decompression with excellent clinical results.

We present the case of a 55-year-old female with right-sided TN due to a petrous meningioma and an adjacent superior cerebellar artery loop compressing the trigeminal nerve. The patient underwent endoscopic tumor resection and MVD, resulting in significant pain relief and improved facial sensation. This case emphasizes the need to address both compressive etiologies in TN cases associated with CPA tumors to achieve the best clinical outcomes.

**Supplementary Video** Endoscopic resection of right petrous meningioma with microvascular decompression of the superior cerebellar artery loop compressing the trigeminal nerve.


